# Trends in Arthritis Prevalence and Associated Chronic Health Indicators Among Adults: Insights From the Behavioral Risk Factor Surveillance System (BRFSS) Database

**DOI:** 10.7759/cureus.58925

**Published:** 2024-04-24

**Authors:** Blessing Eze, Joshua T Green, Ransford Asante, Okelue E Okobi, Kristine Glory F Mercene, Charles T Ogbodo, Eberechukwu G Anamazobi, Amaka S Alozie

**Affiliations:** 1 Internal Medicine, Creighton University School of Medicine, Phoenix, USA; 2 Surgery, Sibley Memorial Hospital, Washington DC, USA; 3 General Practice, Mother and Child Hospital, Kasoa, GHA; 4 Family Medicine, Larkin Community Hospital Palm Springs Campus, Miami, USA; 5 Family Medicine, Medficient Health Systems, Laurel, USA; 6 Family Medicine, Lakeside Medical Center, Belle Glade, USA; 7 Internal Medicine, Mount Sinai Chicago, Chicago, USA; 8 Internal Medicine, Médecins Sans Frontières (Doctors without Borders), Talata Mafara, NGA; 9 Internal Medicine, American International School of Medicine, McDonough, USA; 10 Internal Medicine, Abia State University, Uturu, NGA

**Keywords:** brfss database, adults, trends, chronic health indicators, prevalence, arthritis

## Abstract

Background

Arthritis is a prevalent, chronic condition with significant implications for morbidity and healthcare utilization. Understanding trends in arthritis prevalence and associated chronic health indicators is vital for informing public health interventions and healthcare policies.

Objective

This retrospective study aimed to analyze trends in arthritis prevalence and associated chronic health indicators among adults using data from the Behavioral Risk Factor Surveillance System (BRFSS) database.

Methods

This retrospective study utilized data from the BRFSS database covering 2019 to 2022. Participants included United States adults aged 18 years and older who completed BRFSS surveys during the specified period. Primary variables included arthritis prevalence and its correlation with chronic health indicators and demographics. Data collection involved standardized telephone questionnaires administered annually, with rigorous attention to data quality and consistency. Prevalence estimates were calculated using weighted proportions, and statistical analysis utilized analysis of variance (ANOVA).

Results

The study revealed relatively stable arthritis prevalence over the study period, with notable demographic variations. Arthritis prevalence remained stable (2019: 43.3%, 2021: 42.5%). Females consistently had higher rates than males (2019: 45%, 2021: 44.9%). Activity limitation, joint pain, and work limitation were more prevalent in arthritis patients. White, non-Hispanic individuals had higher rates than other groups. Physical inactivity increased from 2019 (29.4%) to 2022 (72.4%), particularly in males. Counseling for physical activity was lower in males. Targeted interventions are needed to address these disparities and improve arthritis management.

Conclusion

This study provides insights into trends in arthritis prevalence and associated chronic health indicators among United States adults. The findings underscore the importance of considering demographic factors in arthritis prevention and management strategies. Targeted interventions promoting physical activity counseling, particularly among high-risk populations, are warranted to address the rising trend of physical inactivity among individuals with arthritis.

## Introduction

Arthritis, characterized by joint inflammation, pain, and stiffness, remains a significant public health concern globally, affecting millions of individuals and posing substantial burdens on healthcare systems and economies [1,2]. Arthritis, encompassing various forms such as osteoarthritis, rheumatoid arthritis, and gout, is one of the leading causes of disability worldwide [3].

Its prevalence has steadily increased, partly attributed to aging populations and lifestyle factors such as sedentary behavior and obesity. Arthritis affects a significant portion of the United States population, with over 58 million adults affected, half of whom are of working age. By 2040, it is projected that 78 million United States adults will have arthritis [4]. Imaging studies reveal arthritis in over one-third of Americans, a figure expected to rise due to aging demographics [5]. Osteoarthritis is the most prevalent form, affecting 19-30% of adults over 45 years in the knee, 27% in the hand, and 27% in the hip. The lifetime risk for osteoarthritis is estimated at 40% for men and 47% for women, increasing to 60% with a body mass index (BMI) over 30 [4,6,7].

Arthritis encompasses a spectrum of disorders characterized by inflammation of the joints. With over 100 identified types, arthritis manifests in various forms, each presenting unique symptoms, causes, and treatment approaches. From rheumatoid arthritis to osteoarthritis and lupus arthritis to gout, understanding the classifications and distinctions among these conditions is crucial for effective diagnosis and management [1,2,8,9]. Osteoarthritis (OA) initiates progressive cartilage deterioration, resulting in subchondral cysts, osteophytes, and plate thickening. Inflammatory molecules like interleukin-6 induce proteolytic enzymes, exacerbating joint collagen degradation. Aging exacerbates chondrocyte dysfunction, enhancing OA susceptibility [8]. Rheumatoid arthritis (RA) stems from autoimmune responses, triggering chronic inflammation, endothelial activation, and synovial hyperplasia. [9]. Gout arises from monosodium urate crystallization, inducing IL-1-mediated inflammation and acute arthritis. Pseudogout's calcium pyrophosphate dihydrate crystals exacerbate joint damage, especially in osteoarthritic areas, with metabolic disorders heightening deposition risks [10]. Septic arthritis follows bacterial invasion, stimulating cytokine release, cartilage degradation, and synovial membrane hyperplasia. Staphylococcus aureus predominates, with gram-negative bacteria common in specific conditions [11].

Since there is currently no cure, arthritis management focuses on alleviating symptoms, enhancing function, and improving quality of life. Treatment options encompass medication, non-drug therapies like physical therapy or education, and surgical interventions if warranted. Effective symptom management is crucial for pain reduction, disability prevention, and overall well-being [12]. The Centers for Disease Control and Prevention (CDC) Arthritis Program advocates five key strategies for managing arthritis and its symptoms: acquiring new self-management techniques, maintaining physical activity levels, maintaining open communication with healthcare providers, managing body weight effectively, and implementing joint protection measures. This comprehensive approach aims to empower individuals with arthritis to lead fulfilling lives despite their condition [12,13].

Despite advancements in medical treatment and management strategies, the burden of arthritis remains substantial, exerting a profound impact on individuals, families, and society as a whole. Therefore, a thorough examination of arthritis prevalence and its associated chronic health indicators is crucial for devising effective public health interventions and improving the overall well-being of affected individuals [1,2]. The Behavioral Risk Factor Surveillance System (BRFSS) database provides a rich source of data for monitoring arthritis prevalence and associated trends based on surveyed reported cases and offers valuable insights into the demographic, geographic, and socioeconomic factors influencing its distribution and impact. [14]

This paper aimed to analyze the prevalence of arthritis in adults using 2019-2022 BRFSS data. It explores arthritis's relationship with chronic health indicators like activity limitation, joint pain, work limitation, physical inactivity, counseling, and education participation. Understanding these associations informs public health policy and practice regarding arthritis management.

## Materials and methods

Data source and study design

This retrospective study utilized data from the BRFSS database covering the years 2019 to 2022. BRFSS conducts telephone interviews with adults in the United States, collecting self-reported data on various health behaviors, chronic conditions, and preventive services. Established in 1984, BRFSS is the world's largest continuous health survey system, operating nationwide and covering all states, the District of Columbia, and three territories. With over 400,000 annual adult interviews, it provides a comprehensive overview of health-related behaviors and conditions.

Study participants and inclusion criteria

Participants included United States adults aged 18 years and older who completed BRFSS surveys during the specified period. Inclusion criteria required valid responses on arthritis status, activity limitation, joint pain, work limitation, physical inactivity, counseling, and educational participation. Primary variables of interest encompassed arthritis prevalence and its correlation with various chronic health indicators and demographics, including gender, race/ethnicity, and geography.

Data collection and quality assurance

Data were extracted from BRFSS surveys conducted annually from 2019 to 2022. Each year, the BRFSS conducts over 400,000 adult interviews via telephone. The data gathered by the BRFSS team are then transmitted to the CDC, where they undergo aggregation by state, analysis, and subsequent publication by each state at the end of the year. Our study relied on these publicly available data, which we analyzed to derive our findings. The analysis focused on responses from adults who self-reported arthritis diagnoses. Relevant datasets were systematically retrieved from the database using standardized protocols, and rigorous attention was given to data quality and consistency. Cleaning procedures addressed missing values, outliers, and inconsistencies, ensuring precision in the analysis. The dataset was aggregated by chronic indicators, year, gender, and other pertinent categories to facilitate structured statistical examination.

Data analysis and statistical methods

Prevalence estimates for arthritis and associated chronic health indicators were calculated using weighted proportions to address BRFSS's complex sampling design. Estimates were stratified by demographics such as gender, race/ethnicity, and geographic region to assess disparities and trends over time. Rigorous data preprocessing ensured integrity, including cleaning, validation checks, and imputation of missing values. Descriptive statistics summarized demographic characteristics and arthritis prevalence. Statistical analysis utilized one-way analysis of variance (ANOVA) to examine variations in prevalence rates across demographic attributes. Significance was set at p < 0.05, accounting for complex survey design and utilizing the IBM SPSS Statistics for Windows, Version 27 (Released 2020; IBM Corp., Armonk, New York, United States).

Ethical considerations

It is widely recognized that the Institutional Review Board (IRB) considers the analysis of de-identified, publicly available data that lacks individually identifiable information to not constitute human subject research according to the criteria outlined in 45 CFR 46.102. Therefore, this type of analysis does not require an IRB review.

## Results

Arthritis among adults

The prevalence of arthritis among adults in the United States was examined across gender and race/ethnicity categories. In 2019, the overall age-adjusted prevalence of arthritis was 22.8% (95% confidence interval (CI): 22.6 -23.1), with females reporting a higher prevalence (25.7%, 95% CI: 25.4 - 26.0) compared to males (19.7%, 95% CI: 19.4 - 20.0). By 2021, the prevalence remained stable, but by 2022, there was a significant increase to 23.9% (95% CI: 23.7 - 24.1). Stratification by race/ethnicity revealed notable disparities, with the highest prevalence observed among multiracial individuals in 2022 (32.4%, 95% CI: 30.7 - 34.2). The p-values for overall prevalence differences between gender (p = 0.0003) and racial groups (p < 0.001) were statistically significant, as provided in Table 1 below.

**Table 1:** Age-adjusted prevalence of chronic health indicators (arthritis among adults and activity) -: not reported

Indicator	Overall age-adjusted prevalence	Male	Female	White, non-Hispanic	Black, non-Hispanic	Hispanic	Asian, non-Hispanic	Hawaiian or Pacific Islander, non-Hispanic	American Indian or Alaska Native, non-Hispanic	Multiracial, non-Hispanic
Arthritis among adults (Age-adjusted prevalence 95% CI)										
2019	22.8 (22.6 - 23.1)	19.7 (19.4 - 20.0)	25.7 (25.4 - 26.0)	24.8 (24.5 - 25.1)	23.5 (22.8 - 24.2)	17.2 (16.5 - 17.9)	-	-	29.4 (27.4 - 31.5)	28.9 (27.2 - 30.6)
2021	22.8 (22.6 - 23.10)	19.8 (19.4 - 20.1)	25.6 (25.3 - 26.0)	24.8 (24.5 - 25.1)	24.4 (23.6 - 25.1)	17.5 (16.7 - 18.3)	-	-	28.6 (26.7 - 30.5)	26.4 (24.6 - 28.2)
2022	23.9 (23.7 - 24.1)	21 (20.6 - 21.3)	26.6 (26.2 - 26.9)	25.7 (25.5 - 26.0)	24.9 (24.2 - 25.6)	19.3 (18.5 - 20.1)	12.8 (11.5 - 14.1)	23.2 (19.6 - 27.3)	28.2 (26.0 - 30.4)	32.4 (30.7 - 34.2)
P-value		0.0003		<0.001						
Activity limitation due to arthritis among adults with arthritis										
2019	9.7 (9.5 - 9.8)	7.8 (7.6 - 8.0)	11.4 (11.2 - 11.7)	10.5 (10.3 - 10.7)	10.2 (9.7 - 10.7)	7.3 (6.8 - 7.8)	-	-	-	15.2 (13.9 - 16.7)
2021	9.7 (9.5 - 9.9)	7.7 (7.5 - 8.0)	11.6 (11.3 - 11.8)	10.4 (10.2 - 10.6)	10.9 (10.4 - 11.5)	7.5 (7.0 - 8.1)	-	-	-	13.6 (12.1 - 15.2)
P-value	-	0.02545		<0.001						
Severe joint pain among adults with arthritis										
2019	32.6 (31.7 - 33.6)	27.6 (26.2 - 29.0)	36.2 (35.0 - 37.5)	27.6 (26.6 - 28.6)	48.8 (45.4 - 52.3)	-	-	-	-	-
2021	30.4 (29.5 - 31.4)	25.7 (24.2 - 27.3)	33.8 (32.6 - 35.1)	25.1 (24.1 - 26.1)	46.9 (43.3 - 50.4)	-	-	-	-	-
P-value	-	0.0190		0.0052						

Activity limitation due to arthritis among adults with arthritis

Activity limitation among adults with arthritis was explored, highlighting differences by gender and race/ethnicity. In 2019, 9.7% (95% CI: 9.5 - 9.8) of adults reported activity limitation, with females (11.4%, 95% CI: 11.2 - 11.7) experiencing higher rates compared to males (7.8%, 95% CI: 7.6 - 8.0). By 2021, the prevalence remained relatively stable, but the gender disparity persisted. The p-values for differences in activity limitation prevalence between gender and racial groups were statistically significant (p = 0.02545; p < 0.001). Notably, the prevalence of activity limitation remained relatively stable over the years, with slight fluctuations within the confidence intervals.

Severe joint pain among adults with arthritis

The prevalence of severe joint pain among adults with arthritis was examined, with attention to gender and racial disparities. In 2019, the prevalence of severe joint pain was 32.6% (95% CI: 31.7 - 33.6), with notable variations by race/ethnicity. By 2021, the overall prevalence had slightly decreased to 30.4% (95% CI: 29.5 - 31.4), with significant gender disparities persisting. However, specific data points for severe joint pain in 2022 are not available. The p-values for differences in severe joint pain prevalence between gender groups and racial categories were statistically significant (p = 0.0190; p = 0.0052).

Work limitation due to arthritis among adults aged 18-64 years with arthritis

The prevalence of work limitations due to arthritis among adults aged 18-64 years showed a slight decrease from 2019 to 2021. In 2019, the overall prevalence was 43.3% (95% CI: 42.1 - 44.5), which decreased to 42.5% (95% CI: 41.2 - 43.8) in 2021. When stratified by gender, males had a prevalence of 40.9% (95% CI: 39.1 - 42.7) in 2019 and 39.1% (95% CI: 37.0 - 41.2) in 2021. Conversely, females exhibited slightly higher rates, with a prevalence of 45% (95% CI: 43.4 - 46.5) in 2019 and 44.9% (95% CI: 43.3 - 46.6) in 2021. Specifically, among White, non-Hispanic individuals, the prevalence was 41% (95% CI: 39.7 - 42.3) in 2019, decreasing to 38.6% (95% CI: 37.2 - 40.1) in 2021. The difference in prevalence between genders was not statistically significant (p = 0.10826) (Table 2).

**Table 2:** Age-adjusted prevalence of chronic health indicators (limitation and activity) -: not reported

Indicator	Overall	Male	Female	White, non-Hispanic		Black, non-Hispanic	Hispanic	Asian, non-Hispanic	Hawaiian or Pacific Islander, non-Hispanic	American Indian or Alaska Native, non-Hispanic	Multiracial, non-Hispanic
Work limitation due to arthritis among adults aged 18–64 years with arthritis											
2019	43.3 (42.1 - 44.5)	40.9 (39.1 - 42.7)	45 (43.4 - 46.5)		41 (39.7 - 42.3)	-	-	-	-	-	-
2021	42.5 (41.2 - 43.8)	39.1 (37.0 - 41.2)	44.9 (43.3 - 46.6)		38.6 (37.2 - 40.1)	-	-	-	-	-	-
P-value		0.10826									
Physical inactivity among adults with arthritis											
2019	29.4 (28.6 - 30.2)	27.4 (26.1 - 28.7)	30.9 (29.9 - 31.9)		28.1 (27.2 - 29.1)	35 (32.4 - 37.6)	-	-	-	-	-
2021	28.3 (27.4 - 29.2)	24.6 (23.3 - 26.0)	30.9 (29.7 - 32.1)		26.7 (25.8 - 27.7)	35.3 (32.0 - 38.8)	31.3 (28.4 - 34.4)	-	-	-	-
2022	72.4 (71.6 - 73.3)	76.4 (75.3 - 77.5)	69.6 (68.4 - 70.8)		74.3 (73.4 - 75.1)	68.6 (65.9 - 71.3)	66.5 (63.4 - 69.4)	78 (72.5 - 82.7)	77.4 (68.0 - 84.7)	67.4 (61.8 - 72.5)	73.1 (69.6 - 76.4)
P-value		0.825196907			0.873202799						
Received healthcare provider counseling for physical activity among adults with arthritis											
2019	68.6 (67.6 - 69.5)	62.4 (60.9 - 63.9)	73 (71.8 - 74.1)		67.2 (66.2 - 68.2)	72.8 (69.2 - 76.2)	-	-	-	-	-
2021	67.2 (66.2 - 68.1)	62.8 (61.1 - 64.4)	70.3 (69.2 - 71.5)		66.1 (65.0 - 67.1)	73.3 (70.4 - 76.1)	70.9 (67.7 - 73.9)	-	-	-	-
P-value		0.10798658			0.008793762						
Have taken an educational class to learn how to manage arthritis symptoms among adults with arthritis											
2019	16.4 (15.6 - 17.2)	16.6 (15.3 - 17.9)	16.2 (15.2 - 17.2)		16 (15.1 - 16.9)	-	-	-	-	-	-
2021	15.9 (15.1 - 16.7)	16.4 (15.1 - 17.8)	15.5 (14.5 - 16.6)		15 (14.1 - 15.8)	-	-	-	-	-	-
P-value		0.233750123									

Physical inactivity among adults with arthritis

In 2019, the overall prevalence was 29.4% (95% CI: 28.6 - 30.2). Males exhibited a slightly lower prevalence at 27.4% (95% CI: 26.1 - 28.7), while females had a higher prevalence of 30.9% (95% CI: 29.9 - 31.9). White, non-Hispanic individuals showed a prevalence of 28.1% (95% CI: 27.2 - 29.1), whereas Black, non-Hispanic individuals had a notably higher prevalence at 35% (95% CI: 32.4 - 37.6). In 2021, the overall prevalence remained relatively stable at 28.3% (95% CI: 27.4 - 29.2), with males showing a decrease to 24.6% (95% CI: 23.3 - 26.0) and females maintaining a prevalence of 30.9% (95% CI: 29.7 - 32.1). The prevalence among White, non-Hispanic individuals decreased to 26.7% (95% CI: 25.8 - 27.7), while Black, non-Hispanic individuals showed a similar prevalence of 35.3% (95% CI: 32.0 - 38.8). In 2022, a significant increase in physical inactivity prevalence was observed across all groups.

The overall prevalence rose to 72.4% (95% CI: 71.6 - 73.3), with males and females reporting rates of 76.4% (95% CI: 75.3 - 77.5) and 69.6% (95% CI: 68.4 - 70.8), respectively. White, non-Hispanic individuals had a prevalence of 74.3% (95% CI: 73.4 - 75.1), while Black, non-Hispanic individuals exhibited a prevalence of 68.6% (95% CI: 65.9 - 71.3). There were no statistically significant differences in prevalence between genders (p = 0.825196907) or between racial groups (p = 0.873202799) (Table 2).

Received healthcare provider counseling for physical activity among adults with arthritis

The percentage of adults with arthritis who received healthcare provider counseling for physical activity remained relatively stable over the years. In 2019, the overall prevalence was 68.6% (CI: 67.6 - 69.5). Among males, the prevalence was slightly lower at 62.4% (CI: 60.9 - 63.9), while females had a higher prevalence of 73% (CI: 71.8 - 74.1). White, non-Hispanic individuals exhibited a prevalence of 67.2% (CI: 66.2 - 68.2), and Black, non-Hispanic individuals had a notably higher prevalence of 72.8% (CI: 69.2 - 76.2).

By 2021, there was a slight decrease in the overall prevalence to 67.2% (CI: 66.2 - 68.1). Males experienced a minor increase to 62.8% (CI: 61.1 - 64.4), while females had a prevalence of 70.3% (CI: 69.2 - 71.5). Among racial categories, White, non-Hispanic individuals showed a prevalence of 66.1% (CI: 65.0 - 67.1), whereas Black, non-Hispanic individuals maintained a higher prevalence of 73.3% (CI: 70.4 - 76.1). The difference in prevalence between racial groups was statistically significant (p = 0.0087) (Table 2).

Have taken an educational class to learn how to manage arthritis symptoms among adults with arthritis

Participation in wellness groups to manage arthritis symptoms among adults with arthritis showed a slight decrease from 2019 to 2021. In 2019, the overall prevalence was 16.4% (CI: 15.6 - 17.2). Among males, the prevalence was slightly higher at 16.6% (CI: 15.3 - 17.9), while females had a slightly lower prevalence of 16.2% (CI: 15.2 - 17.2). White, non-Hispanic individuals exhibited a prevalence of 16% (CI: 15.1 - 16.9). By 2021, there was a slight decrease in the overall prevalence to 15.9% (CI: 15.1 - 16.7). Males experienced a slight decrease to 16.4% (CI: 15.1 - 17.8), while females had a slightly lower prevalence of 15.5% (CI: 14.5 - 16.6). White, non-Hispanic individuals showed a prevalence of 15% (CI: 14.1 - 15.8). There were no statistically significant differences in prevalence between genders (p = 0.233) (Table 2).

Based on geography

**Figure 1:**
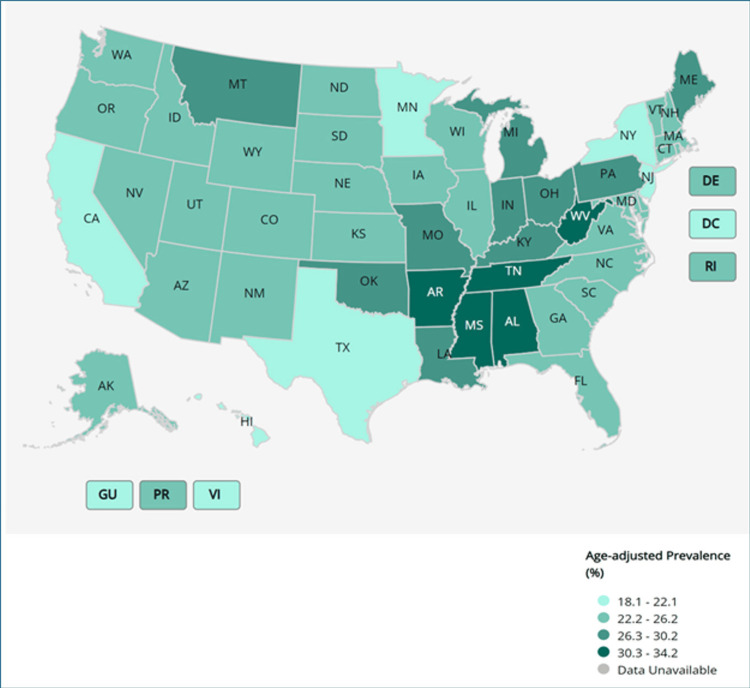
Arthritis among adults: all states, DC, and territories HHS: health and human services; MMWR: Morbidity and Mortality Weekly Report; AL: Alabama; AK: Alaska; AZ: Arizona; AR: Arkansas; CA: California; CO: Colorado; CT: Connecticut; DE: Delaware; FL: Florida; GA: Georgia; HI: Hawaii; ID: Idaho; IL: Illinois; IN: Indiana; IA: Iowa; KS: Kansas; KY: Kentucky; LA: Louisiana; ME: Maine; MD: Maryland; MA: Massachusetts; MI: Michigan; MN: Minnesota; MS: Mississippi; MO: Missouri; MT: Montana; NE: Nebraska; NV: Nevada; NH: New Hampshire; NJ: New Jersey; NM: New Mexico; NY: New York; NC: North Carolina; ND: North Dakota; OH: Ohio; OK: Oklahoma; OR: Oregon; PA: Pennsylvania; RI: Rhode Island; SC: South Carolina; SD: South Dakota; TN: Tennessee; TX: Texas; UT: Utah; VT:  Vermont; VA: Virginia; WA: Washington; WV: West Virginia; WI: Wisconsin; WY: Wyoming

Table 3 below presents the top ten states in the United States with the highest age-adjusted prevalence of arthritis. Overall, the prevalence of arthritis in the United States was 23.9% (95% CI: 23.7 - 24.1). Among these states, West Virginia had the highest prevalence at 34.2% (95% CI: 32.8 - 35.7), followed by Arkansas (30.7%, 95% CI: 29.2 - 32.2) and Mississippi (30.9%, 95% CI: 29.3 - 32.6). Other states with high prevalence rates include Alabama (30.4%, 95% CI: 28.9 - 32.0), Kentucky (30.0%, 95% CI: 28.3 - 31.8), and Tennessee (30.3%, 95% CI: 28.8 - 31.9). These findings highlight regional disparities in arthritis prevalence across the United States (Figure 1).

**Table 3:** Top ten states with the highest age-adjusted prevalence CI: confidence interval

Location	Prevalence % (95% CI)
United States	23.9 (23.7 - 24.1)
Alabama	30.4 (28.9 - 32.00)
Arkansas	30.7 (29.2 - 32.2)
Kentucky	30 (28.3 - 31.8)
Louisiana	28.3 (26.9 - 29.7)
Mississippi	30.9 (29.3 - 32.6)
Ohio	27.8 (26.9 - 28.7)
Oklahoma	27.4 (26.2 - 28.6)
Pennsylvania	28 (26.3 - 29.8)
Tennessee	30.3 (28.8 - 31.9)
West Virginia	34.2 (32.8 - 35.7)

## Discussion

Arthritis is a prevalent chronic condition affecting millions of adults worldwide, leading to significant morbidity and healthcare utilization. Understanding trends in arthritis prevalence and associated chronic health indicators is crucial for informing public health interventions and healthcare policies aimed at mitigating its burden.

The findings of this study revealed notable trends in arthritis prevalence over the study period. Overall, the prevalence remained relatively stable, with minor fluctuations observed between 2019 (22.8%) and 2022 (23.9%). However, further analysis revealed variations in prevalence among demographic subgroups. Notably, females consistently exhibited higher arthritis prevalence compared to males across all years, which is consistent with previous epidemiological studies [15-17]. In previously published National Health Interview Survey data, age-standardized arthritis prevalences were higher among women (20.9%) than men (16.3%) [16]. Similar trends were reported by a population-based study, where the overall age-adjusted and sex-adjusted annual RA incidence in 2005-2014 was 53/100,000 in women and 29/100,000 in men [17]. The reasons for this gender disparity warrant further investigation and may include hormonal, genetic, and socio-cultural factors influencing arthritis susceptibility and reporting.

The study identified demographic disparities in arthritis prevalence, with significant variations observed among racial/ethnic groups. White, non-Hispanic individuals consistently reported higher arthritis prevalence compared to other racial/ethnic groups, while Black, non-Hispanic individuals exhibited elevated prevalence rates compared to the overall population. These findings align with a previously conducted study that reported that age-standardized arthritis prevalences were higher among non-Hispanic White (20.1%) than among Hispanic or Latino (14.7%) or non-Hispanic Asian adults (10.3%) [16]. In another cross-sectional study conducted by Carpenter et al., the arthritis prevalence rate was 25.1% for Whites, 24.7% for Blacks, and 15.7% for Latinos [18,19]. These disparities underscore the importance of considering sociodemographic factors in arthritis prevention and management strategies.

In addition to arthritis prevalence, this study examined associated chronic health indicators, including activity limitation, joint pain, work limitation, physical inactivity, counseling, and educational participation. Understanding the relationship between arthritis and these indicators provides insights into the multifaceted nature of the condition and its impact on individuals' daily functioning and quality of life. Notably, individuals with arthritis reported higher rates of activity limitation, joint pain, and work limitation compared to those without arthritis, highlighting the substantial burden imposed by the condition on functional capacity and productivity [20]. Similar findings were reported by Hootman et al., where a survey conducted in 2010-2012 found that 22.7% of all adults had doctor-diagnosed arthritis and 9.8% had arthritis-attributable activity limitation. By 2040, the number of US adults with doctor-diagnosed arthritis is projected to increase by 49%, and the number of adults with arthritis-attributable activity limitations will increase by 52% [4].

Physical inactivity is a modifiable risk factor for arthritis progression and associated comorbidities. This study observed concerning trends in physical inactivity among adults with arthritis, with a notable increase in prevalence during the study period. This finding is consistent with a previous study, which also reported a rising trend in physical inactivity among individuals with arthritis over a similar period [4,21]. Interestingly, males consistently exhibited higher rates of physical inactivity compared to females. This suggests potential gender-specific barriers to engaging in regular physical activity among individuals with arthritis, which warrants further investigation.

Moreover, disparities in counseling for physical activity were observed, with lower rates reported among males compared to females. This is in line with the results of a study by Duca et al., which found gender disparity in receiving counseling for physical activity [22]. Collectively, these findings highlight the need for targeted interventions promoting physical activity counseling, particularly among high-risk populations, and emphasize the importance of addressing gender disparities in healthcare delivery.

Educational classes play a crucial role in empowering individuals with arthritis to manage their symptoms effectively and improve their self-management skills. However, this study found relatively low participation rates in educational classes among adults with arthritis, with no significant change observed over time. Barriers to participation, including access, awareness, and perceived benefits, may contribute to the underutilization of educational resources among individuals with arthritis [22]. Addressing these barriers through targeted outreach efforts and innovative educational programs tailored to individuals' needs may enhance participation and improve health outcomes.

Several limitations should be considered when interpreting the findings of this study. Firstly, the use of self-reported data from telephone surveys may be subject to recall bias and social desirability bias, potentially impacting the accuracy of prevalence estimates. Additionally, the cross-sectional nature of the study limits the ability to establish causality or temporal relationships between arthritis prevalence and associated factors. Future research should employ longitudinal designs to elucidate the dynamic interplay between arthritis and its determinants over time. Furthermore, exploring the impact of environmental, psychosocial, and behavioral factors on arthritis prevalence and management outcomes may provide valuable insights into effective intervention strategies.

## Conclusions

This study provides valuable insights into trends in arthritis prevalence and associated chronic health indicators among United States adults. Gender and race/ethnicity analysis revealed significant disparities in arthritis prevalence, activity limitation, severe joint pain, work limitation, physical inactivity, and healthcare counseling. While overall prevalence remained stable, certain groups experienced disparities. Addressing these gaps requires targeted interventions promoting physical activity, education, and healthcare access. Regional differences highlight the need for tailored public health strategies. By addressing contributing factors, policymakers and healthcare providers can improve arthritis management and quality of life nationwide. Our findings emphasize the importance of equitable healthcare access and contribute valuable insights into arthritis epidemiology. Further research and targeted interventions are needed to address the complex factors impacting arthritis prevalence and its consequences.
